# RAPD PCR Profile, Antibiotic Resistance, Prevalence of *armA* Gene, and Detection of KPC Enzyme in *Klebsiella pneumoniae* Isolates

**DOI:** 10.1155/2018/6183162

**Published:** 2018-02-04

**Authors:** Arezoo Saadatian Farivar, Jamileh Nowroozi, Gita Eslami, Azar Sabokbar

**Affiliations:** ^1^Department of Microbiology, Karaj Branch, Islamic Azad University, Karaj, Iran; ^2^Department of Microbiology, Islamic Azad University North Tehran Branch, Tehran, Iran; ^3^Department of Microbiology, School of Medicine, Shahid Beheshti University of Medical Sciences, Tehran, Iran

## Abstract

The increasing prevalence of multidrug-resistant *Klebsiella pneumoniae* strains isolated from hospitals shows the limitation of recent antibiotics used for bacterial eradication. In this study, 81 *K. pneumoniae* isolates were collected from three hospitals in Tehran. Antibiotic susceptibility test showed the highest rates of resistance to cefotaxim (85.5%) and ceftazidime (78.3%), and the lowest rates of resistance were detected for colistin (16.9%), streptomycin (16.8%), and chloroamphenicol (21.7%). Eleven different resistance patterns were observed. Sixty-six out of 81 isolates (81.5%) were found to be multidrug resistant (MDR), and 35.8% of them belonged to A3 resistance pattern. 7.4% and 66.7% were KPC enzyme and *armA* gene positive, respectively. RAPD PCR assay of these bacteria showed 5 clusters, 16 single types, and 14 common types, and there was not any correlation between genetic patterns of the isolates and presence of resistance agents. Simultaneous detection of resistance-creating agents could be an important challenge for combination therapy of MDR *K. pneumoniae*-caused infections.

## 1. Introduction

In the 1980s, gram-negative pathogens were successfully defeated using cephalosporins, carbapenems, and fluoroquinolones [1]. *K. pneumoniae* is one of the most widespread nosocomial pathogens causing urinary tract infections, bacteremia, and pneumonia in different parts of the world [2, 3]. Extensive use of antibiotics and thereby the development of various resistance mechanisms have led to the emergence of MDR (multidrug resistance) *K. pneumoniae* [4]. KPC- (*Klebsiella pneumoniae* carbapenemase-) producing *K. pneumoniae* strains are the most common carbapenamase-producing pathogens worldwide [5] that have also been reported in Iran in many studies [6–8]. Seventeen different types in the KPC family have been reported to date [9], among which one family is located on a conjugative plasmid that encodes resistance to gentamicin and tobramycin [10]. The *armA* gene, which is responsible for resistance to the majority of the aminoglycosides and is the most prevalent in Asia [11], was found to be located on the same plasmid of KPC-producing strains reported formerly from Italy [12], China [13], and Poland [14]. Coexistence of the resistance-inducing agents can result in the emerging of MDR *K. pneumoniae* strains. Epidemiological characterization of MDR *K. pneumoniae* can help to prevent the spread of resistant strains [15]. RAPD PCR (random amplification of polymorphic DNA PCR) is a simple, rapid, inexpensive, and widely used typing method which does not require advanced knowledge of DNA sequences of the target organism. RAPD typing has been successfully employed for epidemiological analysis of many bacteria [16] as well as clinical isolates of *K. pneumonia* [17].

This study aimed to detect the RAPD PCR fingerprint, antibiotic resistance patterns, and the presence of *armA* gene in clinical KPC-producing isolates and nonproducing isolates of *K. pneumoniae*.

## 2. Materials and Methods

### 2.1. Bacterial Collection

From September 2015 to March 2016, clinical samples were collected from both inpatients and outpatients attending three hospitals in Tehran, Iran. The samples were transferred to the microbiology laboratory, and biochemical tests were performed to identify *K. pneumoniae*. The pure isolates were stored at −20°C in Trypticase soy broth containing 20% glycerol.

### 2.2. Susceptibility Testing and Phenotypic Detection of KPC

The antimicrobial susceptibility test was conducted using the Kirby–Bauer disk diffusion method on Mueller–Hinton agar (Merck, Germany). Also, for colistin-resistant isolates, MIC was determined through a broth microdilution method, according to the Clinical and Laboratory Standard Institute (CLSI) guidelines. The isolates were tested for susceptibility to the following 16 antimicrobial agents (MAST Group, Merseyside, UK): ofloxacin (OFX, 5 *μ*g), ciprofloxacin (CIP, 5 *μ*g), norfloxacin (NOR, 10 *μ*g), gentamicin (GM, 10 *μ*g), amikacin (AK, 30 *μ*g), streptomycin (HLS, 10 *μ*g), tobramycin (TOB, 10 *μ*g), kanamycin (K, 30 *μ*g), ticarcillin (TIC, 75 *μ*g), cefotaxim (CTX, 30 *μ*g), ceftazidime (CAZ, 30 *μ*g), azetreonam (ATM, 30 *μ*g), imipenem (IMI, 10 *μ*g), trimethoprim (TR, 5 *μ*g), chloramphenicol (C, 30 *μ*g), and colistin (CO, 10 *μ*g). *Escherichia coli* ATCC 25922 was used as a control strain. Also, detection of KPC enzyme was performed by the modified Hodge test [18].

### 2.3. Detection of armA Gene

Bacteria were cultured in Luria–Bertani (LB) broth medium and were incubated overnight at 37°C. DNA was extracted from *K. pneumoniae* isolates by using Genomic DNA Isolation Kit (Global Gene Network Bio, cat. no. k-3000, South Korea) according to the manufacturer's instruction. The presence of *armA* gene was evaluated using PCR technique with the *armA*-F: TCGGAACTTAAAGACGACGA and *armA*-R: CCATTCCCTTCTCCTTTCCA (designed in this study) sequences. The PCR product was analyzed by electrophoresis in a 1% (W/V) agarose gel in TBE buffer at 95 V for 45 min, stained with ethidium bromide, and observed under UV lighting using Gel Doc. Then, DNA sequencing was performed by Bioneer Company (Korea), blasted in NCBI, and analyzed by Finch TV software.

### 2.4. RAPD PCR

Molecular typing of *K. pneumoniae* isolates was performed using RAPD PCR analysis with the primer 640 (CGTGGGGCCT) (Faza Biotech, Tehran, Iran). Reaction mixtures (25 *µ*l) contained: 12.5 *µ*l (Exprime Taq Premix (2X) Mastermix (Global Gene Network Bio, cat. no. G-5000)), 7.5 *µ*l Distilled water, 200 pmol of primer, and 3 *µ*l DNA template. The program used was as follows: 5 min at 94°C followed by 36 cycles of 1 min at 94°C, 1 min at 36°C, and 2 min at 72°C followed by a final extension at 72°C for 9 min. PCR products were then electrophoresed on 1% agarose gels and visualized by ethidium bromide staining. To determine the similarity rate among the isolates, they were analyzed by the unweighted pair-group method with arithmetic mean (UPGMA) using GelClust software.

## 3. Results

### 3.1. Bacterial Isolation

Eighty-one *K. pneumoniae* were isolated from the patients (44 males and 37 females). Samples were isolated from different clinical specimens including urine, 49 (60.5%); blood cultures, 16 (19.7%); wounds, 5 (6.2%); sputum, 4 (5%); intra-abdominal, 3 (3.7%), and others 4 (5%). The hospital ward distribution was as follows: pediatric ward, 24 (29.6%); outpatient, 18 (22.2%); intensive care unit, 10 (12.3%); surgical unit, 6 (7.4%); infection unit, 2 (2.5%); and others, 21 (26%).

### 3.2. Determination of Antibiotic Resistance and KPC Enzyme Detection

Antibiogram showed that the resistance rates of the isolates were as follows: ofloxacin 65%, ciprofloxacin 68.7%, norfloxacin 66.3%, gentamicin 66.3%, amikacin 51.8%, tobramycin 56.7%, kanamycin 79.5%, ticarcillin 82%, streptomycin 16.9%, cefotaxim 85.5%, ceftazidime 78.3%, azetreonam 79.5%, imipenem 45.8%, trimethoprim 74.7%, chloramphenicol 21.7%, and colistin 16.9%. One colistin-resistant isolate had a MIC of 8 *µ*g/ml, and the other all had MIC values of 4 *µ*g/ml. All the colistin-resistant isolates except one of them belonged to one hospital. Five colistin-resistant *K. pneumoniae* exhibited resistance to imipenem of which one was positive for KPC production. The antibiotic resistance pattern of the isolates is shown in Table 1. The highest rate of the resistance belonged to the A9 pattern (64.2%) which found to be resistant to three flouroquinolons. Also, the lowest rate of resistance belonged to the A4 pattern (4.9%) which was resistant to ciprofloxacin, ofloxacin, norfloxacin, imipenem, amikacin, and colistin. MDR is generally determined as resistance to three or more classes of antibiotics [19]. In the present study, 66 out of 81 isolates were resistant to 3 or more classes of antibiotics; thereby, 81.5% of the isolates were found to be MDR. Also, the modified Hodge test showed that 6 isolates (7.4%) were positive for KPC production with 3 resistance patterns (Table 2).

### 3.3. Molecular Detection

The PCR showed that 54 isolates (66.7%) of the isolates had *armA* gene. Aminoglycoside resistance patterns in *armA* positive isolates were presented in Table 3.

### 3.4. RAPD PCR

**Figure 1:**
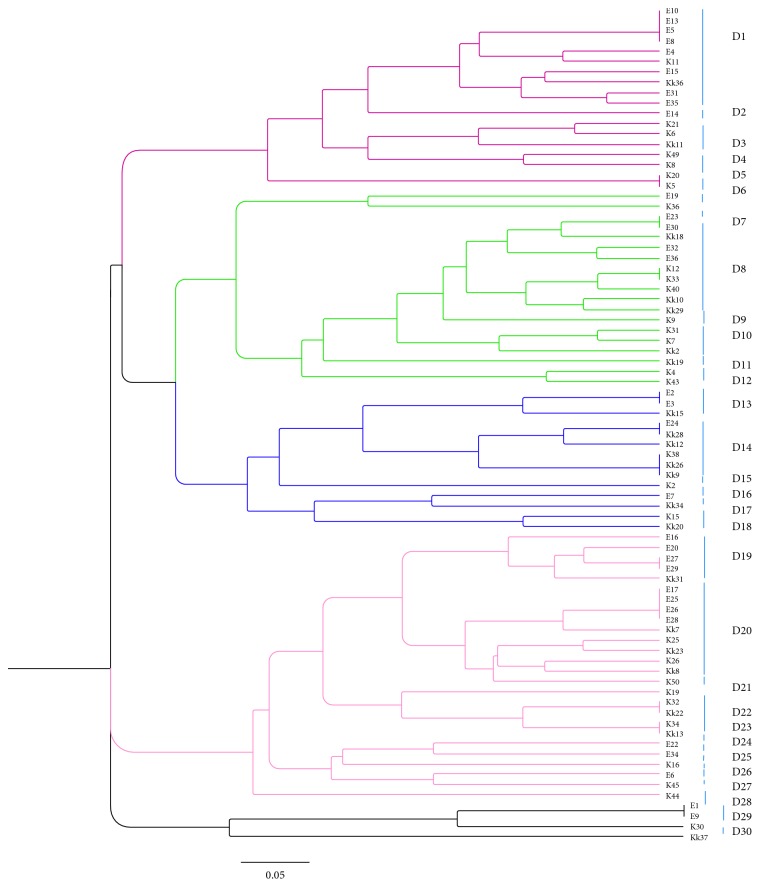
Cluster analysis of *Klebsiella pneumoniae* based on RAPD typing.

Genetic analysis of RAPD showed 30 distinct patterns from D1 to D30, as 5 distinct clusters (Figure 1). The information of isolates is shown in Table 4. The isolates were considered as the same pattern if the level of similarity was ≥85%. The numbers of the isolates in each cluster (from cluster 1 to 5) were 18, 19, 14, 26, and 4, respectively. The highest redundancy belonged to pattern D1, D8, and D20. There was not any correlation between genetic patterns of the isolates and presence of *armA* gene or KPC production. The isolates which showed 100% similarity, belonging to different clusters, all found to have *armA* gene.

## 4. Discussion

In the present study, 81 *K. pneumoniae* were isolated from clinical samples. Antibiotic resistance patterns and their relationships among different clonal isolates were investigated. Eleven antibiotic patterns were found (A1–A11), showing much diversity in the resistance patterns. In the study by Hassan et al. [16] and Ben-Hamouda et al. [17], different antibiotic resistance patterns were reported, too [20]. In the present study, 81.5% were MDR. The presence of multidrug resistance community acquired *K. pneumoniae* highlights the need for accurate planning to control and prevent of the dissemination of MDR strains. Most of the KPC-producing isolates harbored *armA* gene and were resistant to carbapenems, aminoglycosides, and fluoroquinolones. According to our previous study, Real Time PCR showed an increased expression level of OqxAB and AcrAB efflux pumps in fluoroquinolone-resistant isolates in comparison with the sensitive ones (data not shown). So, the role of efflux pump in creating fluoroquinolone-resistant strains could be identified in these isolates [21]. Also, the aminoglycoside resistance rates suggested 16S rRNA methylase activity. In the present study, approximately 70% of the aminoglycoside-resistant strains carried the *armA* gene as Zhou et al. reported [22]. Therefore, some of these *K. pneumoniae* isolates have three features of resistance: KPC, efflux pumps, and *armA* gene. So they can be resistant to fluoroquinolones, cephalosporins, carbapenems and a spectrum of aminoglycosides as well. These strains can turn out to an important challenge for community and hospital officials by disseminating among the patients in hospitals and making the treatment process more difficult. Coexistence of the active efflux pump, *armA* gene, and KPC enzymes in *K. pneumoniae* can help to resist against the combination therapy. This hypothesis is also recommended by Zacharczuk et al. [14] and Jiang et al. [13] who observed KPC production and *armA* gene in clinical isolates of *K. pneumoniae*.

Furthermore, about 17% of the isolates were resistant to colistin, among which all but one isolate had MIC 4 *µ*g/ml. Although colistin-resistant isolates were related to five different clusters, 42.9% of them belonged only to the fourth cluster from one hospital, indicating a genetically specific circulating cluster. Colistin is considered as effective treatment against MDR and carbapenem-resistant bacteria, such as *K. pneumoniae*, but resistance to this agent has begun to emerge. So, more studies to determine the best treatment for infections caused by resistant *K. pneumoniae* are needed. In the present study, genotyping analysis showed different genetic patterns among pathogenic *K. pneumoniae* isolates. Lim et al. [23], Ben-Hamouda et al. [17], Pai et al. [24], and Eftekhar and Nouri [25] performed separately the genotyping of clinical *K. pneumoniae* strains and showed that they were genetically diverse and heterogeneous. Therefore, this tool has got the ability to identify related and unrelated isolates. The correlation between the antibiotic resistance patterns and RAPD analysis demonstrated that different genetic patterns had different antibiotyping profiles. Also, KPC-producing *K. pneumoniae* were found to belong to different clusters, and the results of RAPD PCR implicated that there is no correlation between the genetic patterns and presence of *armA* gene or KPC-producing *K. pneumoniae*, as Ma et al. [26] reported that nonclonally spread of ESBL-producing *K. pneumoniae* strains with *armA* or *rmtB* mediates aminoglycoside resistance. It seems that because most resistance genes are carried by the mobile genetic elements, they can easily transmit among the bacteria.

Of importance, the low number of the isolates was one of our limitations. Collecting more clinical isolates, using more powerful discriminating typing methods such as PFGE and analysis of *armA* and *bla*_kpc_ genes' expression level may improve the quality of our results in the following studies.

## 5. Conclusion

Although fluoroquinolones seem to be a good choice for treating *Klebsiella* infections, several studies have shown increasing resistance of *K. pneumoniae* isolates to these agents. Aminoglycosides have been considered as an adequate therapeutic against both gram-negative and gram-positive pathogens and also combination therapy with *β*-lactams and aminoglycosides is well accepted for the treatment of the systemic infections caused by *K. pneumoniae*, so simultaneous detection of resistance creating agents to fluoroquinolones, *β*-lactams, and aminoglycosides in these strains is of clinical importance. Emphasis on the suitable use of antibiotics, effective infection control measures, and identification of antibiotic resistance mechanisms by molecular procedures are necessary to reduce the incidence of infections caused by antibiotic-resistant organisms.

## Figures and Tables

**Table 1:** Antibiotic resistance patterns.

Resistance pattern number	Number of isolates (%)	Antibiotics
A1	11 (13.6)	OFX, NOR, CIP, IMI, K, GM, AK, TOB, HLS
A2	19 (23.4)	OFX, NOR, CIP, IMI, GM, AK, TOB, K
A3	29 (35.8)	OFX, NOR, CIP, IMI, K, GM
A4	4 (4.9)	CIP, OFX, NOR, IMI, AK, CO
A5	12 (14.8)	AK, GM, TOB, K, HLS
A6	25 (30.9)	CIP, OFX, NOR, IMI, AK
A7	33 (40.7)	CIP, OFX, NOR, IMI
A8	30 (37)	CIP, OFX, NOR, AK
A9	52 (64.2)	CIP, OFX, NOR
A10	11 (13.6)	TR, TIC, C
A11	25 (30.9)	IMI, AK

OFX: ofloxacin; NOR: norfloxacin; CIP: ciprofloxacin; IMI: imipenem; GM: gentamicin; AK: amikacin; TOB: tobramycin; HLS: streptomycin; K: kanamycin; CO: colistin; TR: trimethoprim; TIC: ticarcillin; C: chloramphenicol.

**Table 2:** Antibiotic resistance patterns and presence of *armA* gene.

Isolate name	Resistance pattern	*armA*
1	OFX, NOR, CIP, IMI, K, GM, AK, TOB, HLS	+
2	OFX, NOR, CIP, IMI, K, GM, AK, TOB	+
3	OFX, NOR, CIP, IMI, K, GM, AK, TOB	−
4	OFX, NOR, CIP, IMI, K, GM	−
5	OFX, NOR, CIP, IMI, K, GM	+
6	OFX, NOR, CIP, IMI, K, GM	+

OFX: ofloxacin; NOR: norfloxacin; CIP: ciprofloxacin; IMI: imipenem; K: kanamycin; GM: gentamicin; AK: amikacin; TOB: tobramycin; HLS: streptomycin.

**Table 3:** Aminoglycoside resistance in *armA* positive isolates.

Aminoglycosides	Resistant number (%)	*armA *+ number (%)
Kanamycin	66 (79.5)	46 (69.7)
Tobramycin	47 (56.7)	32 (68)
Gentamicin	55 (66.3)	38 (69)
Amikacin	43 (51.8)	32 (74.4)
Streptomycin	14 (16.9)	12 (87.7)

**Table 4:** Analysis of strains with their RAPD profiles.

Isolate number	*armA*	KPC	Date	Origin	Department	Hospital	RAPD pattern
1	−	−	February 2016	Urine	Gynecology and obstetrician	A	D1
2	−	−	February 2016	Urine	Outpatient	A	D1
3	−	−	February 2016	Urine	Outpatient	A	D1
4	−	−	February 2016	Urine	Laparoscopy	A	D1
5	−	−	February 2016	Urine	Outpatient	A	D1
6	+	−	September 2015	Wound	Others	B	D1
7	−	−	February 2016	Urine	Outpatient	A	D1
8	+	−	October 2015	Blood	Pediatric	C	D1
9	−	−	February 2016	Urine	Rheumatology	A	D1
10	+	−	March 2016	Urine	Nephrology	A	D1
11	−	−	February 2016	Urine	Others	A	D2
12	+	−	December 2015	Blood	Hematology	B	D3
13	+	−	January 2015	Wound	Surgery	B	D3
14	+	−	January 2015	Urine	Pediatric	C	D3
15	−	+	November 2015	Urine	Outpatient	B	D4
16	−	−	November 2015	Blood	Infectious	B	D4
17	+	−	September 2015	Intraabdominal	Gastrointestinal	B	D5
18	+	+	September 2015	Sputum	ICU	B	D5
19	−	−	February 2016	Urine	Cancer	A	D6
20	+	−	October 2015	Sputum	Bone marrow transplantation	B	D7
21	−	−	February 2016	Urine	Dermatology	A	D8
22	−	+	February 2016	Urine	Cardiology	A	D8
23	+	−	January 2015	Blood	Pediatric	C	D8
24	−	−	March 2016	Urine	Outpatient	A	D8
25	−	−	March 2016	Urine	Outpatient	A	D8
26	+	−	January 2015	Blood	ICU	B	D8
27	−	−	January 2015	Sputum	ICU	B	D8
28	+	−	December 2015	Urine	Outpatient	B	D8
29	+	−	December 2015	Blood	Pediatric	C	D8
30	+	−	December 2015	Blood	Pediatric	C	D8
31	+	−	October 2015	Wound	Surgery	B	D9
32	+	−	November 2015	Intraabdominal	Cancer	B	D10
33	−	−	October 2015	Others	NICU	B	D10
34	+	−	November 2015	Blood	Pediatric	C	D10
35	+	−	December 2015	Urine	Pediatric	C	D11
36	+	−	September 2015	Sputum	ICU	B	D12
37	+	−	October 2015	Wound	Surgery	B	D12
38	+	−	March 2016	Urine	ICU	A	D13
39	+	+	March 2016	Urine	Others	A	D13
40	+	−	January 2015	Blood	Pediatric	C	D13
41	+	−	February 2016	Urine	Outpatient	A	D14
42	+	−	January 2015	Others	Pediatric	C	D14
43	+	−	January 2015	Urine	Pediatric	C	D14
44	+	−	December 2015	Others	Outpatient	B	D14
45	+	+	December 2015	Urine	Pediatric	C	D14
46	+	−	December 2015	Blood	Pediatric	C	D14
47	−	−	September 2015	Urine	Surgery	B	D15
48	−	−	February 2016	Urine	Outpatient	A	D16
49	+	−	October 2015	Urine	Pediatric	C	D17
50	+	−	November 2015	Urine	Endocrinology	B	D18
51	+	−	November 2015	Blood	Pediatric	C	D18
52	−	−	March 2016	Urine	Cardiology	A	D19
53	+	−	March 2016	Urine	Others	A	D19
54	−	−	March 2016	Urine	Pediatric	A	D19
55	−	−	March 2016	Urine	Outpatient	A	D19
56	−	−	January 2015	Urine	Pediatric	C	D19
57	−	−	February 2016	Urine	Pediatric	A	D20
58	−	−	February 2016	Urine	Surgery	A	D20
59	−	−	February 2016	Urine	ICU	A	D20
60	−	−	February 2016	Urine	Others	A	D20
61	+	−	November 2015	Urine	Pediatric	C	D20
62	+	−	November 2015	Urine	Outpatient	B	D20
63	+	−	January 2015	Urine	Pediatric	C	D20
64	+	−	November 2015	Blood	Outpatient	B	D20
65	+	−	January 2015	Urine	Pediatric	C	D20
66	+	−	January 2015	Others	Outpatient	B	D20
67	+	−	September 2015	Wound	Others	B	D21
68	+	−	November 2015	Blood	Hematology	B	D22
69	+	−	November 2015	Blood	Pediatric	C	D22
70	+	−	September 2015	Urine	NICU	B	D22
71	+	−	September 2015	Urine	Pediatric	C	D22
72	+	−	March 2016	Urine	Surgery	A	D23
73	+	−	March 2016	Urine	Cardiology	A	D24
74	+	−	December 2015	Blood	NICU	B	D25
75	−	−	February 2016	Urine	Pediatric	A	D26
76	+	−	October 2015	Urine	Outpatient	B	D27
77	+	−	December 2015	Urine	ICU	B	D28
78	−	−	February 2016	Urine	Outpatient	A	D29
79	−	−	February 2016	Urine	Outpatient	A	D29
80	+	+	January 2015	Intraabdominal	Infectious	B	D29
81	+	−	November 2015	Blood	Pediatric	C	D30

## Abbreviations

RAPD: Random amplification of polymorphic DNA
KPC: *Klebsiella pneumoniae* carbapenemase
PCR: Polymerase chain reaction
CLSI: Clinical and Laboratory Standards Institute
MDR: Multiple drug resistance
NCBI: National Center for Biotechnology Information
ESBL: Extended-spectrum beta-lactamases
PFGE: Pulsed field gel electrophoresis.
